# Kenaf Fiber-Reinforced Biocomposites for Marine Applications: A Review

**DOI:** 10.3390/ma18050999

**Published:** 2025-02-24

**Authors:** Yang Huang, Mohamed Thariq Hameed Sultan, Farah Syazwani Shahar, Andrzej Łukaszewicz, Zbigniew Oksiuta, Rafał Grzejda

**Affiliations:** 1 Department of Aerospace Engineering, Faculty of Engineering, Universiti Putra Malaysia, Serdang 43400, Selangor, Malaysia; huangyang821210@hotmail.com (Y.H.); farahsyazwani@upm.edu.my (F.S.S.); 2 College of Engineering and Architecture, Beibu Gulf University, Qinzhou 535011, China; 3 Laboratory of Biocomposite Technology, Institute of Tropical Forestry and Forest Products (INTROP), Universiti Putra Malaysia, Serdang 43400, Selangor, Malaysia; 4 Aerospace Malaysia Innovation Centre (944751-A), Prime Minister’s Department, MIGHT Partnership Hub, Jalan Impact, Cyberjaya 63000, Selangor, Malaysia; 5 Institute of Mechanical Engineering, Faculty of Mechanical Engineering, Bialystok University of Technology, 45C Wiejska St., 15-351 Bialystok, Poland; 6 Institute of Biomedical Engineering, Faculty of Mechanical Engineering, Bialystok University of Technology, 45C Wiejska St., 15-351 Bialystok, Poland; z.oksiuta@pb.edu.pl; 7 Faculty of Mechanical Engineering and Mechatronics, West Pomeranian University of Technology in Szczecin, 19 Piastow Ave., 70-310 Szczecin, Poland; rafal.grzejda@zut.edu.pl

**Keywords:** kenaf fiber, fiber-reinforced composite, physicochemical properties, mechanical properties, manufacturing technique, biocomposites, marine application

## Abstract

Fiber-reinforced composites are widely utilized across various industries, including aerospace, automotive, and marine, due to their outstanding mechanical properties and lightweight characteristics. Natural fibers, as promising reinforcements, have the potential to replace synthetic fibers in certain areas to meet the growing demand for environmental protection and sustainability. These biocomposites offer numerous benefits, including reduced carbon footprints, diminished reliance on non-renewable resources, and increased natural biodegradability. In addition, utilizing such eco-friendly materials is a critical strategy for balancing industry progress and environmental protection. Kenaf fiber, a superior bast fiber known for its excellent mechanical properties and high cellulose content, presents considerable advantages for enhancing the performance of biocomposites. This review explores the potential of kenaf fiber-reinforced biocomposites for marine applications, focusing on their fabrication and testing methods to evaluate their physicochemical and mechanical properties. This paper examines the chemical composition and mechanical properties of the kenaf fiber, investigates the excellent performance advantages of kenaf fiber-based biocomposites by hybridization manufacturing, and provides an overview of the status and challenges of applying such biocomposites in marine environments. Based on this review, it is evident that kenaf fiber-reinforced biocomposites have significant superiority for marine applications with the advancement of manufacturing techniques.

## 1. Introduction

**Figure 1:**
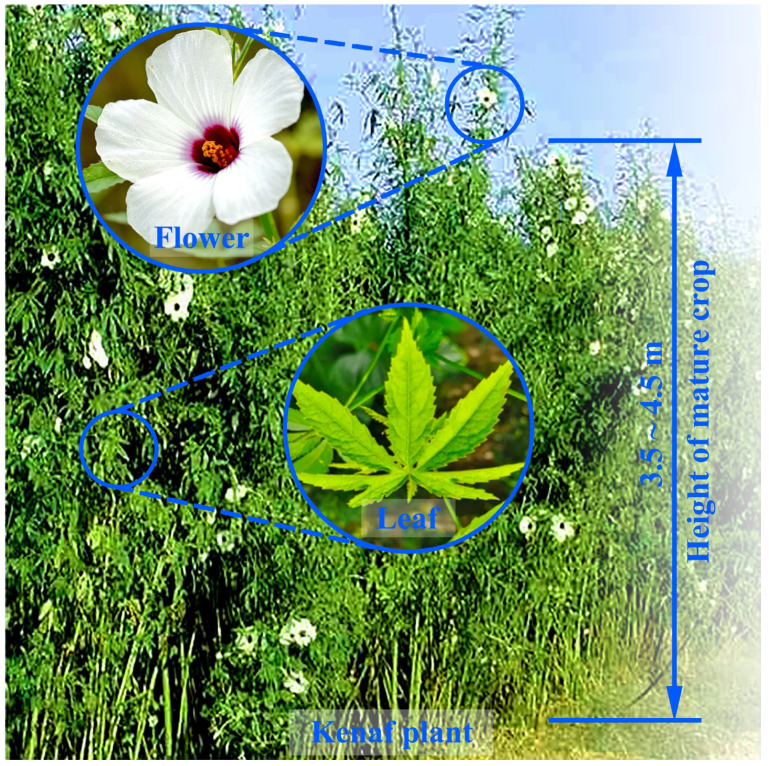
Appearance of the kenaf plant at maturity [9].

Kenaf (*Hibiscus cannabinus* L.) is an annual herbaceous plant belonging to the Malvaceae family [1]. This crop has a long cultivation history, tracing back to 4000 BC in ancient Africa, and demonstrates remarkable environmental adaptability [2,3,4]. The growing recognition of the economic and environmental benefits of kenaf is prompting an increase in its cultivation worldwide. The plant has been extensively distributed and successfully cultivated in various Asian and American nations, especially in regions characterized by high rainfall and plentiful solar radiation, including China, Malaysia, Thailand, and India [5]. In addition, kenaf is an exceptionally resilient crop with excellent capability to grow in a range of climatic conditions and soil environments [6]. Normally, the dicotyledon crop can grow rapidly from 3.5 to 4.5 m in height in a short time [7]. With its rapid growth cycle and considerable plant height, this plant achieves a remarkable yield, producing approximately three to four times more than traditional forests [8]. Kenaf has multiple advantages, such as rapid growth, high yield, and extreme adaptability. Its leaves are palmately lobed, and the flowers are large and beautiful, usually yellow or white with a red center. A mature kenaf plant is shown in Figure 1. 

Traditionally, kenaf has been utilized as livestock feed and rope production material for a long period. However, the applications of kenaf have expanded considerably with advances in science and processing technology, and its production includes fabrics, pulp, biological composites, bioenergy, seed oils, medicines, and medical devices [4,10,11]. Moreover, kenaf is a significant food crop and valuable medicinal plant containing multiple bioactive compounds that can potentially provide health benefits to both humans and animals [1]. In addition, it is an economical and eco-friendly cellulose resource that can provide high-quality cellulose for human use at a low cost [6]. Furthermore, kenaf is a commercial crop with environmentally beneficial properties. It has a notable positive contribution to reducing the carbon footprint, with a carbon dioxide absorption capacity that is approximately 4–5 times more significant than that of trees [8]. Kenaf, an economically valuable crop, has attracted worldwide attention. For example, the Malaysian government envisions kenaf as the nation’s third key economic crop, following rubber and oil palm [12]. The worldwide crop yield will be analyzed in Section 2. In previous expectations, the global kenaf market scale is expected to exceed $854 million by 2025 [12]. The dual positive trend of expanding market demand and increasing economic value will further promote the development of the kenaf-relevant application. Kenaf fiber-reinforced composite materials have become a popular research field, and their related products have been applied in the transportation industry.

Kenaf thrives in environments characterized by high temperatures and ample moisture [12]. The crop yield is usually between 11 and 18 tons/ha, which is related to the kenaf variety, sunshine length, maturity, sowing, and harvesting time [13]. Additionally, a better yield can be obtained under a plant density of 20–40 plants/m^2^, and an irrigation water supply of 300–400 mm was recorded in prior research [14]. Kenaf production has a low energy consumption of 15 MJ per kilogram, which is only a quarter of the energy required to produce glass fibers [15]. 

The kenaf stalk consists of two major parts: an outer thin cortical layer (bast) and an inner ligneous part (core). Bast constitutes 35–40% of the total stem weight and consists mainly of long fibers, while core constitutes 60–65% of the total stem weight and consists mainly of short fibers [3]. Therefore, the plant has a high bast kenaf fiber yield. The kenaf fiber, being a soft bast fiber, has many superior properties, such as strong mechanical properties, excellent moisture absorption, and rapid water dispersal [16]. Kenaf plays an important role in providing new and renewable industrial fibers, especially in many developing countries such as India, China, Malaysia, etc. [1]. Kenaf fiber, as an important natural soft fiber, has received extensive attention [17,18]. In addition, kenaf fiber can be employed as an alternative to synthetic and other natural fibers [19], which are widely applied in the field of fiber-reinforced composites utilized in aerospace, automotive, and civil engineering applications [20,21,22].

Fiber-reinforced composites are made from two or more separate raw materials, such as reinforcements, matrixes, and fillers, with different properties [23,24]. These raw materials are tightly combined through complex chemical and physical effects to form a new type of material with unique features [25]. These composite materials can be designed and manufactured according to application requirements, such as light weight, high strength, high modulus, corrosion resistance, and other advantages utilized in various environments [26,27,28,29]. The traditional reinforcements of fiber-reinforced composites are synthetic fibers, such as glass fiber, carbon fiber, and aramid fiber, which have good capabilities to enhance fiber-reinforced composites [30,31]. However, the production process of synthetic fibers consumes a lot of energy and non-renewable resources; additionally, these synthetic fibers are difficult to degrade naturally and completely and will also increase the environmental burden of landfill disposal [32,33]. 

Both environmental protection and sustainable development have led to research into alternatives to synthetic fiber materials, and natural fibers are the best substitutes in this field [34]. Environmentally friendly biocomposites have attracted increasing attention. In addition, these biocomposites, which are made from renewable and biodegradable natural materials, have positive benefits in decreasing carbon footprint and resource sustainability [35]. However, using natural fibers to fabricate composites requires overcoming challenges, such as the susceptibility of plant fibers in wet states, the instability of natural fiber features, and the difficulty of compatibility between hydrophilic vegetable fibers and hydrophobic polymers [2,36].

This review focuses on the current research on kenaf fibers and their composites to explore their potential for marine applications. First, the review overviews the structural and chemical composition of kenaf fiber, focusing on its characteristics, mechanical properties, extraction methods, and potential application prospects of kenaf fiber. Subsequently, the mechanical and physical properties of the kenaf fiber-reinforced biocomposites will be evaluated, including their tensile, flexural, compressive, and impact strengths. In addition, this review also summarized the ASTM test standards applicable to the use of composites and the commonly adopted manufacturing processes for fiber composites. Finally, this article outlines the status of marine applications of kenaf fiber-reinforced biocomposites, and the challenges they face in marine application environments.

## 2. Kenaf Fiber

Generally, kenaf fiber, an essential raw material across a wide range of industries, is a bast fiber derived from the phloem of the kenaf plant [8]. The stem of the kenaf plant has a thick bast layer. Therefore, the kenaf bast fiber yield is very high, contributing to the proportion of the bast layer weight of 35% of the total dry weight of the stem. Additionally, the height of the mature kenaf plant is considerable, facilitating the extraction of longer kenaf fiber with excellent features [37]. Furthermore, cultivating kenaf crops has a positive effect on the environment due to kenaf being a highly efficient photosynthetic plant. During the growth process, it can absorb large amounts of carbon dioxide from the air and contribute positively to the carbon sink [7]. 

The higher cellulose content of the kenaf fiber endows the fiber with superior mechanical properties and makes the fiber favored than other fibers employed in composites and other industrial applications [37]. In addition, kenaf fiber has excellent synergistic and hybrid capabilities with synthetic and other natural fibers for fabricating composites [38,39]. These composites are popularly utilized for their advantages, such as lightweight, low cost, and high specific mechanical properties [40]. These factors have facilitated the widespread cultivation of kenaf plants globally, with India and China being the major producers of kenaf [37]. As shown in Table 1, the global production of kenaf and related fibers has remained relatively stable, fluctuating slightly at around 200 kilotons in recent years. Developing countries dominate the production of kenaf and related fibers, contributing to over 95% of the global supply. In the 2021/22 period, India and China produced 100 and 41.43 kilotons, accounting for 49.38% and 20.46% of the global production, respectively [4]. This is primarily attributed to the favorable environment and climate for kenaf growth, along with broader application markets [3,4].

### 2.1. Structural and Chemical Composition of Kenaf Fiber

**Figure 2:**
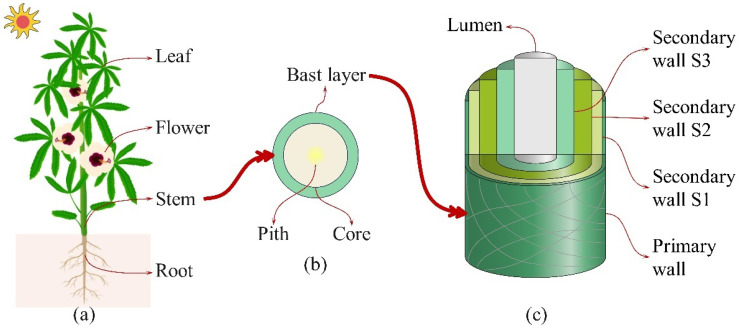
Schematic diagram of the appearance of the kenaf plant and its fiber structure: (**a**) appearance of the kenaf plant; (**b**) cross-section of the stem (collected from the plant); (**c**) structure of kenaf fiber (extracted using chemical or physical methods).

The components of plant fiber are associated with plant cell walls, are the most abundant renewable resource on earth, and are widely used for food, feed, fuel, and fiber [41]. The plant cell wall is an important and intricate structure that provides support and protection to cells. The structure generally consists of a primary cell wall, intermediate layer, and secondary cell wall [42]. Plant cell walls primarily consist of polysaccharides, such as cellulose, hemicellulose, lignin, and pectin [43]. However, the proportional distribution of these constituents varies between the primary and secondary cell walls. In addition, variations in the proportions of polysaccharide constituents among different plant species significantly influence the morphology and properties of plant fibers [44]. Figure 2 illustrates the extraction location of kenaf fiber and its complex multilayer structure. The structure is similar to that of the cell wall, consisting of a thin primary wall and three layers of secondary walls. 

The secondary walls are wrapped inside the primary wall [45]. Both the primary and secondary walls contain cellulose, hemicellulose, and lignin. The secondary cell wall has a significantly higher content of cellulose, hemicelluloses, and lignin than the primary wall; however, the pectin is nearly absent in the secondary wall, and the reason is that pectin is more abundant in the middle lamella of cell walls, and its content decreases gradually from the primary wall toward the interior direction [42]. The natural fibers’ properties are significantly affected by the chemical composition of plant fibers, especially the content of cellulose, hemicellulose, and lignin [46]. Moreover, among the secondary walls, the thickness of the secondary wall (S2) reaches 80% of the total wall thickness of the cellulose fibers and contributes significantly to the mechanical properties of plant fibers [47]. Researchers have reported that the physical properties, chemical composition, and fiber diameter of natural fibers are influenced by factors such as the growth environment and extraction methods, resulting in variations in kenaf fibers [29,48,49]. Ben Mlik et al. investigated the enzymatic and chemical extraction of kenaf fibers and found that chemical extraction breaks the cellulose chains, damaging the fiber structure. In contrast, enzymatically extracted fibers maintain better structural integrity and exhibit properties comparable to those obtained through chemical extraction [50]. Table 2 lists the chemical components of kenaf fibers. In the table, the content of cellulose is in the range of 31% to 72%, hemicellulose is in the range of 13.59% to 23%, lignin is in the range of 6% to 20.1%, pectin is in the range of 0.6% to 9.3%, moisture is in the range of 6.2% to 12%, and ash is in the range of 0.8% to 5%, and the range refers to the minimum and maximum values of different chemical contents. Additionally, the effect of fiber bundles with a lower coefficient of variation can reduce the adverse impact of performance differences in individual natural fibers [48].

### 2.2. Physical and Mechanical Properties of Kenaf Fiber

The physical and mechanical properties of plant fibers are key indexes for evaluating fiber materials, especially when fabricating lightweight and high-strength composite materials [59]. Density is the ratio of mass to volume and is a crucial parameter for mass-sensitive structures (e.g., transportation). Using lightweight materials can effectively reduce the weight of vehicles and improve their maneuverability [60,61]. Tensile strength, tensile modulus, and elongation are commonly used to evaluate the mechanical properties of fibers [62]. These values measure the ability of materials to carry loads, resist deformation, and exhibit toughness [63]. Table 3 lists the typical physical and mechanical properties of the kenaf fibers. From the table, the tensile strength is in the range of 223 MPa to 1193 MPa, the tensile modulus is in the range of 11 GPa to 60 GPa, the elongation at break is in the range of 1.6% to 5.7%, the density is 1.2 g/cm^3^ to 1.5 g/cm^3^, the diameter is 50 μm to 144.8 μm for kenaf fiber, the specific strength is 149 N·m/g to 794 N·m/g, and specific stiffness is 1066.7 N·m/g to 2916.7 N·m/g. Here, the property range of kenaf fiber is established by the minimum and maximum values of the data. The data reveal that the results reported by the researchers are not entirely consistent. The values of the mechanical properties have significant fluctuations. These variations are primarily due to differences in kenaf fiber types, locality of growth, and initial extraction methods [64]. Additionally, irregular variations in the cross-sectional area of natural fibers along the fiber length direction further contribute to these distinctions [65]. Kenaf fibers are outstanding lignocellulosic materials with high specific strength and stiffness, making them ideal for lightweight composite applications. Their use enables structural weight reduction while maintaining the mechanical strength and deformation resistance.

### 2.3. Extraction Methods for Kenaf Fiber

**Figure 3:**
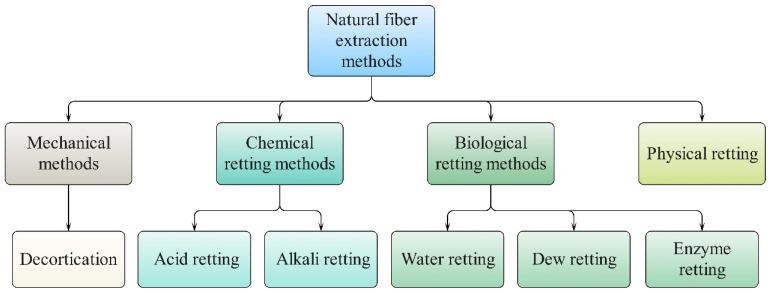
Schematic diagram of natural fiber extraction methods.

The quality of kenaf fiber is significantly influenced by the methods employed during its harvesting and extraction processes [67]. Harvesting kenaf plants is an important part of agricultural production and has a significant effect on the yield and quality of the subsequent fiber extraction [70]. Currently, there are two types of harvesting methods: manual and mechanical methods [71]. Local productivity levels, operational costs, and demands for subsequent processing methods are key factors influencing the selection of a suitable harvesting method. Similarly, the choice of a suitable fiber extraction method plays a critical role in influencing the quality of natural fiber products and their final applications [49]. As illustrated in Figure 3, there are four main retting methods: mechanical, chemical, biological, and physical methods. These methods are further categorized based on the medium utilized, and their principles, advantages, and disadvantages are listed in Table 4. The selection of an appropriate retting method and parameters is determined by the fiber type and its desired performance characteristics.

The diameter and surface morphology of kenaf fibers are affected by the extraction method. The water retting method produces the finest kenaf fibers compared to the mechanical and chemical methods, in the range of 3.2 to 3.7 Tex [77]. Similar results were reported by Amel et al., who also found that the water retting method can produce kenaf fibers with higher tensile strength, reaching 426.1 MPa, which is 8.4% higher than that of the NaOH retting method and 10.2% higher than that of the decorticated method [78]. However, the traditional method of soaking kenaf stems in water takes a long time, typically 2–3 weeks [79]. In addition, Rozyanty et al. compared the effects of water retting and mechanical retting on kenaf bast fibers and their composites [80]. They demonstrated that the water environment provides relatively stable retting conditions for the growth and activity of bacteria, facilitating the production of more uniform, cleaner, and smoother kenaf fibers. These fibers exhibit reduced mechanical damage and can form stronger bonds with the matrix, thereby improving water absorption resistance and mechanical performance. Additionally, compared with mechanically retted kenaf fibers, water-retted kenaf fibers contain fewer hydroxyl groups, further reducing the water uptake of the composites [80]. Soatthiyanon and Crosky extracted kenaf fiber using HNO_3_ and H_2_O_2_/CH_3_COOH extraction methods and found that both methods could remove lignin, pectin, and wax and increase the cellulose crystallinity of kenaf fiber. In the research, they also found that employing H_2_O_2_/CH_3_COOH extraction methods can significantly increase fiber thermal resistance and reduce retting time [81]. Therefore, different extraction methods can be employed depending on the fiber property requirements [82].

## 3. Characteristics of Kenaf Fiber-Reinforced Biocomposites

**Figure 4:**
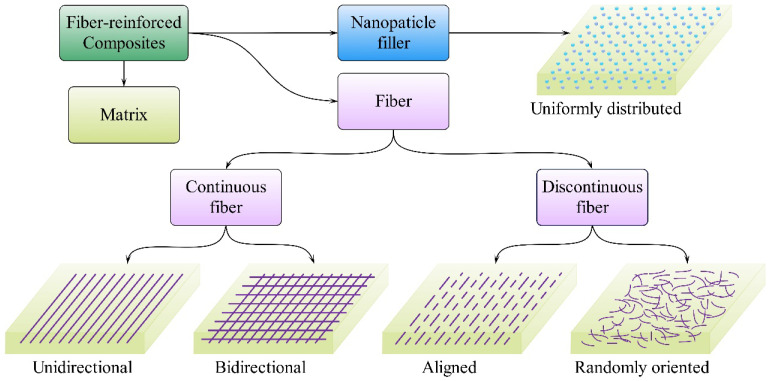
Schematic representation of fibers and nanoparticles distribution in composites.

Fiber-reinforced composites (FRCs), an advanced composite material, are made of fiber reinforcements, polymers, and other fillers using advanced manufacturing techniques [83,84,85]. The distribution of these components in the composite is shown in Figure 4. However, traditional composites typically employ synthetic fibers as reinforcements, such as glass fiber, carbon fiber, and aramid fiber. [86]. These synthetic fibers use non-renewable resources and large amounts of energy in manufacturing processes, creating environmental shocks [32,33]. Landfills are currently the most common and cheapest method of disposing of non-biodegradable materials at the end of their useful life; however, this has negative impacts on ecosystems [87]. Therefore, the utilization of renewable resources for the creation of high-performance industrial materials and products is growing around the globe due to environmental and sustainability concerns [88,89,90]. Plant fibers are becoming increasingly popular in composites because they are environmentally friendly and economical compared to synthetic fibers [91]. Kenaf fiber has a high cellulose content and superior mechanical properties compared with other natural fibers, and has excellent potential to replace synthetic fibers in several special applications [38,92]. Therefore, the application of kenaf fiber can promote the development of sustainable manufacturing processes [19]. Furthermore, utilizing cellulose fibers aligns with the goals of modern industry by promoting green sustainability and contributing to net-zero carbon emissions through carbon offsetting, thereby combating global warming [93]. 

The mechanical properties of kenaf composites are affected by several factors, such as fiber length, fiber mass, fiber orientation, fiber volume fraction, and fiber-matrix interfacial bonding quality [19,94]. High-quality interfacial bonding promotes effective collaboration between fibers and the matrix, enabling an optimized stress distribution and ultimately enhancing the mechanical performance of composite materials. Kenaf fiber-reinforced biocomposites exhibit significant mechanical properties, which will be discussed in Section 5. Kenaf fiber has excellent compatibility with other fibers for the production of hybrid fiber-reinforced biocomposites. Biocomposites reduce the use of fossil fuel resources and have good ecological balance properties [95]. In addition, hybrid composites fabricated by combining two or more fiber types exhibit unique material properties that are often difficult to find in the natural world [19]. Kenaf and glass fibers can be perfectly combined to create superior composites [38]. For instance, Nadzri et al. utilized kenaf and glass fabrics to create hybrid sandwich laminates. After conducting low-velocity impact tests, they found that the laminate made of outer glass fabric and inner kenaf fabric (G/K/G) with a polyester resin matrix offered superior impact resistance up to 10 J [96]. In addition, applying kenaf fiber to manufacture fiber-reinforced composites offers several advantages, such as lightweight properties, reduced cost, and promotion of the development of biological composite materials. The cost of kenaf fiber is significantly lower than that of synthetic fibers, at a quarter of the cost of glass fiber [15]. Compared with glass fiber, kenaf fiber exhibits a density advantage, with its density being only 56% of that of glass fiber [38]. In addition, the biodegradability of kenaf fiber-based composites helps alleviate environmental burdens, making them a more sustainable alternative [97]. Hazrol et al. developed kenaf/cornhusk fiber-reinforced corn starch biocomposites and conducted soil burial tests, revealing that the biocomposites can fully degrade in soil within 10 days [56]. These ecological and environmental benefits have promoted the development of kenaf-fiber-based biocomposites. 

## 4. Composites Testing Using ASTM Standards

The unique ingredients and ingenious structural formations of composite materials make them significantly different from conventional materials in terms of parameters such as strength, durability, stability, and environmental resistance [98,99,100]. In addition, the versatility of composites allows them to be customized for diverse applications. Compared with traditional materials, advanced composites demonstrate exceptional weight reduction capabilities and superior performance under specific conditions, making them highly suitable for advanced engineering applications, including aerospace, automotive, and maritime manufacturing [101,102,103]. Moreover, the composites’ mechanical and physical properties play a critical role in establishing their application potential and ensuring the structural stability and safety of the product. Therefore, composite-specific criteria and procedures must be used to test these materials to determine the essential performance characteristics of composites. Rigorous testing in compliance with ASTM standards is critical for evaluating performance metrics, such as tensile strength, flexural strength, compressive strength, impact resistance, fatigue properties, density, void content, hardness, and water absorption [104]. Table 5 lists the routine test items, corresponding criteria, and obtained data, which are essential for evaluating the material suitability for structural applications. The formulas used for calculating the tensile and flexural properties and water absorption of the composite materials are displayed in the following equations. These results can guide engineers and researchers in optimizing material configurations and selecting appropriate materials for specific engineering applications.

The ultimate tensile strength is calculated as follows:

(1) $$\sigma_{tu}=\frac{P_{max}}{A}$$

where: $\sigma_{tu}$ = ultimate tensile strength, MPa; $P_{max}$ = maximum loading force before break, N; *A* = cross-section area, mm^2^, 

The flexural stress and modulus are calculated as follows:

(2) $$\sigma_{f}=\frac{3PL}{2bt^{2}}$$

(3) $$E=\frac{L^{3}m}{4bt^{3}}$$

where: $\sigma_{f}$ = outer layer stress at loading point, MPa; *P* = loading force, N; *L* = support span, mm; *b* = width of sample, mm; *t* = thickness of sample, mm; *E* = flexural modulus of elasticity, MPa; *m* = Slope of the initial straight-line section of the load-deflection curve, N/mm.

The water absorption is calculated as follows:

(4) $$WaterAbsorption\left(\%\right)=\frac{W_{t}-W_{0}}{W_{0}}\times 100$$

where: $W_{0}$ = initial dry weight of the sample before immersion, g; $W_{t}$ = wet weight of sample at aging time *t*, g.

Accurate testing of the properties of composite materials is fundamental for evaluating their strength and stiffness. Employing appropriate standards, methods, and equipment ensures reliable results, which are critical for assessing the performance of polymer composites in diverse applications. In addition, selecting an appropriate testing scheme and process requires careful consideration of the unique properties and characteristics of the composite material to ensure accurate and meaningful results are obtained. The critical features of composites include dimensional specifications, raw material composition, manufacturing techniques, overall material structure, and intended application scenarios. Furthermore, ensuring an adequate number of test samples for each testing program is essential, containing no fewer than five samples for each group or meeting the minimum requirement specified by the relevant standards. Moreover, the testing environment should be carefully controlled to ensure data reliability, precision, and accuracy.

## 5. Manufacturing and Properties of Kenaf-Based Biocomposites 

Fiber-reinforced composites have a wide range of applications, and their demand continues to increase [106]. However, non-biodegradable synthetic fibers, especially glass fibers, dominate the market share [37]. In response to the environmental concerns linked to the widespread use of non-biodegradable materials, researchers around the world are exploring sustainable alternatives [107]. Among these strategies, the use of natural fibers stands out as a highly viable, cost-effective, and environmentally responsible solution, offering significant benefits in terms of reducing ecological impact [108,109,110]. Increasing the proportion of plant fibers in materials to substitute synthetic ones while ensuring structural safety represents a commendable environmental practice and offers significant opportunities for advancing the application of plant fibers. Biocomposites based on plant fibers have attracted considerable attention from researchers [88,89]. To optimize the reinforcing performance of natural fibers in composite materials, pre-treatment is a crucial step that helps eliminate impurities from the fiber surface, thereby improving the bonding between the fibers and the matrix [111]. In addition, pre-treating the surface of natural fibers before use improves their compatibility and bonding ability of the natural fibers with the matrix and increases the thermal stability of the composites [112]. There are three primary categories of pre-treatments, including physical treatment (heat, laser, plasma, etc.), chemical treatment (alkali, silane, grafting agent, etc.) [113], and biological treatment (fungal and enzyme) [114]. 

As an important chemical surficial modification method, alkali treatment is effective for altering kenaf fiber properties and morphology and influencing the performance of their composites (shown in Table 6). The data in Table 5 indicate that soaking kenaf fibers in a 5–6% sodium hydroxide solution at room temperature for 4 to 8 hours produces the desired effect. Sodium hydroxide (NaOH) reacts with the hydroxyl groups (OH) on the fiber surface, reducing their content and releasing water molecules, while also dissolving hemicellulose, lignin, pectin, and the waxy layer [114]. Additionally, this treatment exposes more cellulose on the fiber surface, thereby increasing the number of reactive sites available for bonding with the matrix [15]. During this process, the plant fiber diameter changes due to the removal of hemicellulose and lignin, which enhances the fiber surface characteristics for better bonding with the matrix [115]. Typically, alkali treatment can increase the surface roughness of plant fibers, which enhances the mechanical interlocking between the fibers and the matrix. However, treatment parameters, such as concentrate, immersion time, and solution temperature, significantly affect the mechanical and morphological properties of kenaf fibers and their composites [66]. Long sock times and high chemical concentrations may increase the potential for fiber structure damage, which could result in poor performance [66,116,117].

### 5.1. Manufacturing Methods for Fiber-Reinforced Composites

The production of fiber-reinforced composite materials is a highly specialized and complex process [37]. This process requires precise control over numerous parameters, including the ratio of fiber-to-matrix, equipment pressure, and fabrication temperature, to ensure that the final material attains the desired mechanical and physical properties. A systematic evaluation of the product size, geometry, and performance specifications is essential for selecting the optimal combination of reinforcing fibers, matrix materials, and manufacturing techniques [69]. Traditional production methods remain widely favored by many manufacturers due to their low cost, ease of operation, and minimal equipment requirements [121].

**Figure 5:**
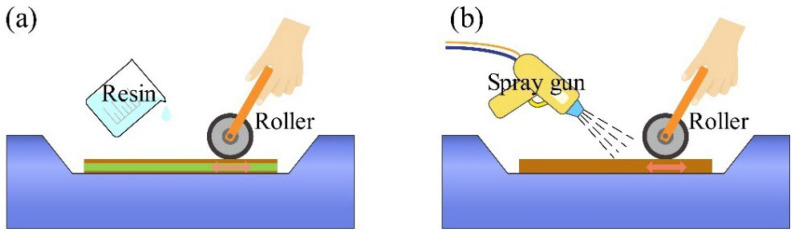
(**a**) Hand lay-up method. (**b**) Spray molding.

The hand lay-up method is the most widely used open-mold manufacturing process for composite materials. This technique is straightforward and does not require sophisticated equipment. The process involves squeezing resin into the fabrics using a roller to ensure thorough impregnation, promote material combination, and eliminate air bubbles, as shown in Figure 5a. Spray molding (SM) exhibits similarities to the hand lay-up technique, particularly in employing a roller to promote the bonding of materials. The primary difference is the use of a spray gun, which sprays a mixture of fibers and resin onto the mold surface using a jet technique. This approach is more automated than the traditional hand lay-up technique, making it particularly suitable for producing large-scale structural components with improved efficiency [69], as shown in Figure 5b.

**Figure 6:**
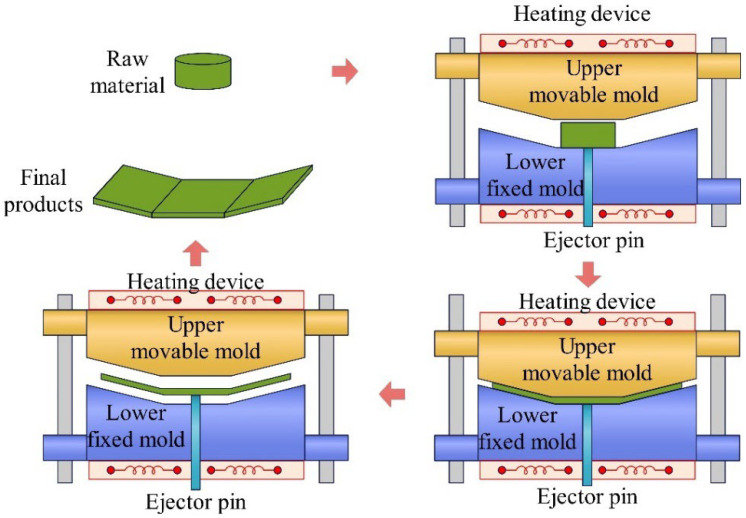
Compression molding.

Compression molding (CM) involves placing raw materials or stacked laminates into a metal mold cavity designed to satisfy specific requirements. Hydraulic equipment then closes the mold and applies a constant pressure, forcing the material to conform to the cavity shape. When heat is applied during the compression phase, the process is referred to as hot-press molding, which is widely applied in processes to fabricate thermoplastic composites, as exhibited in Figure 6.

**Figure 7:**
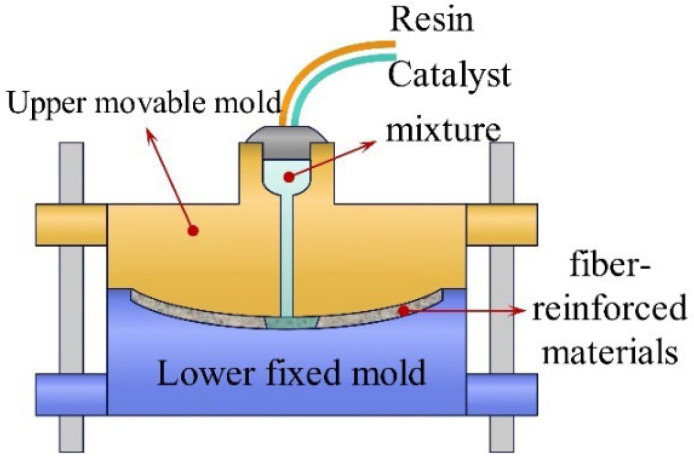
Resin transfer molding.

Resin transfer molding (RTM) is employed to manufacture high-performance composite components. In this process, the liquid resin mixture is injected into a closed mold cavity, where the resin completely permeates the reinforcement material under pressure, filling the entire cavity, as shown in Figure 7. This advanced manufacturing technique allows the creation of various fiber material configurations, including intricate three-dimensional reinforcement structures, making it ideal for fabricating complex configuration materials.

**Figure 8:**
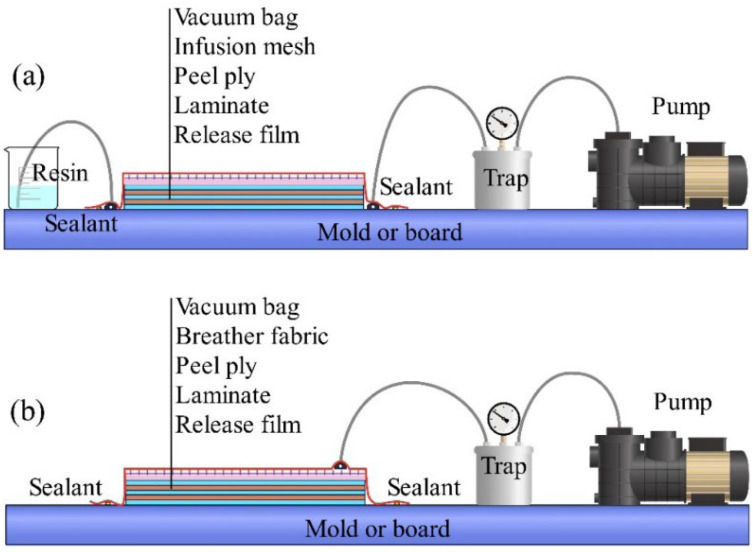
(**a**) vacuum-bagging molding. (**b**) vacuum-assisted resin transfer molding.

The vacuum-bagging technique, as shown in Figure 8a, is often employed as a post-processing step immediately following the hand lay-up process. This method involves isolating the composite component from external air using a plastic film and sealant, followed by vacuuming the interior using a pump to produce denser materials. Employing vacuum bagging can effectively minimize the porosity of the material and improve resin impregnation. If a higher pressure is required, autoclave molding (AM) can be employed, as this method can apply pressures of up to 5 bar. AM is particularly effective in manufacturing composites with superior properties [69]. 

Vacuum-assisted resin transfer molding (VARTM), as shown in Figure 8b, is an advanced technique derived from vacuum-bagging technology. During processing, a vacuum pump creates a pressure differential that drives the resin to flow into and impregnate the reinforcement material. The positions of the resin inlets and outlets play a critical role in the manufacturing process. Achieving complete and efficient impregnation requires careful consideration of core parameters, such as equipment performance, resin viscosity, operating time, injection distance, and resin flow path.

**Figure 9:**
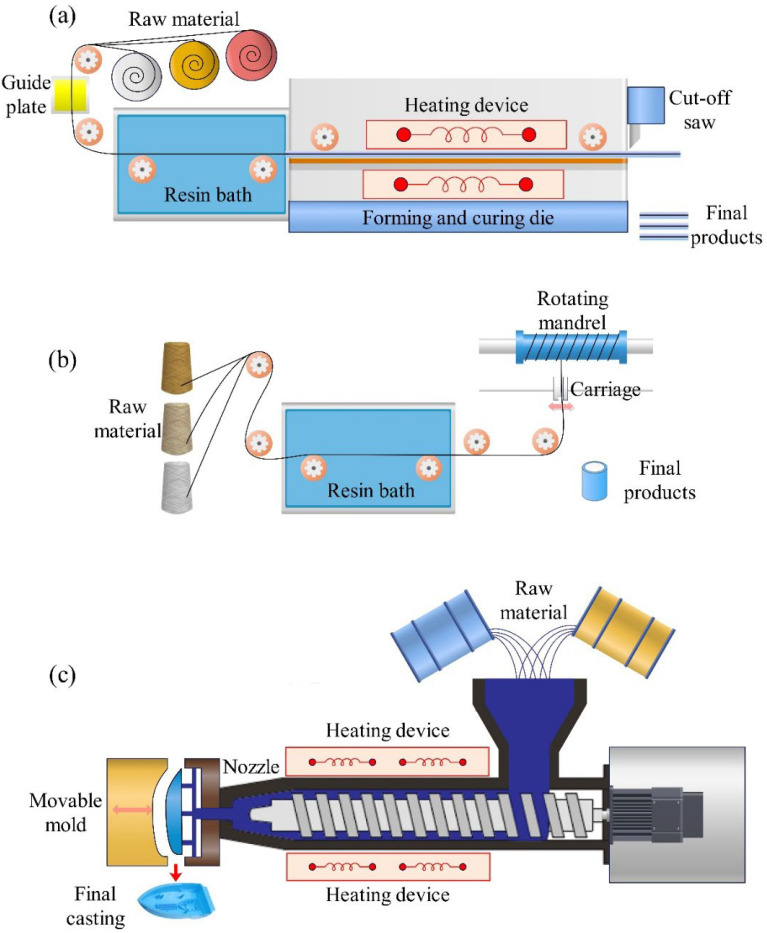
(**a**) Pultrusion process. (**b**) Filament winding process. (**c**) Injection molding.

The pultrusion process, as shown in Figure 9a, is a continuous manufacturing technique that is ideal for producing composite materials with uniform cross-sections and extended lengths. The process involves drawing continuous fibers through a resin bath, curing them in a heated die, and cutting the final product into desired dimensions. This method is well suited for automated industrial production.

The filament winding (FW) process, as shown in Figure 9b, involves the continuous impregnation of fibrous materials with resin, followed by the automated fabrication of hollow composite structures using various winding techniques, including spiral, circumferential, and polar winding. During the filament winding process, the tension of the fibers generates pressure within the unconsolidated fiber layers, reducing the void level of the component [122]. This method can be used for automated industrial production.

Injection molding (IM), as shown in Figure 9c, involves preparing the composition of the fibers and matrix material, which is then injected into the mold cavity to form the composite. This method is particularly advantageous for producing precise components with shorter production cycles, making it highly suitable for large-scale industrial applications.

### 5.2. Properties of Kenaf Fiber-Reinforced Biocomposites

The mechanical properties of composite materials include tensile, compressive, and flexural characteristics, energy absorption capacity, and other performance metrics. Generally, the fiber type and orientation, polymer type, interfacial bonding, and initial microcracks significantly impact the material properties of fiber-reinforced composites by affecting the stress transfer mechanisms and energy absorption efficiency [123,124]. The failure mechanisms of fiber-reinforced composites primarily involve fiber fracture, matrix cracking, and fiber-matrix interface debonding, which often interact under applied loads, ultimately leading to their structural failure. Observable failure molds typically include fiber fracture, fiber bending, fiber pull-out, fiber detachment, interfacial debonding, matrix void formation, matrix crack initiation and propagation, and plastic deformation [108]. Table 7 summarizes the mechanical properties of the kenaf fiber-reinforced composites reported in recent studies. In the table, the tensile strength is in the range of 8.76 MPa to 410.6 MPa, the tensile modulus is in the range of 0.47 GPa to 25.54 GPa, the flexural strength is in the range of 15.7 MPa to 401.0 MPa, and the flexural modulus is in the range of 1.35 GPa to 32.67 GPa. The broad range of values indicates the potential of kenaf fiber-based composites for various applications. The maximum stress values correspond to composites hybridized with synthetic fibers, emphasizing the effectiveness of hybridization technologies in enhancing the mechanical properties of these composites. In addition, employing symmetrical structural configurations and structural styles that use synthetic fibers on the outer layer significantly contributes to making a positive effect on improving the mechanical strength of composites. However, combining kenaf fibers with fibers exhibiting weak mechanical properties leads to composites with significantly reduced tensile and flexural strengths. For instance, the tensile and flexural strengths of kenaf/Areca composites decreased by up to 72.54% and 63.57%, respectively [125]. Moreover, epoxy resin systems are extensively employed as matrix materials in composites due to their low shrinkage and minimal residual stress, significantly enhancing the overall strength of the composites [38,126,127].

Traditionally, natural fiber composites are often used in interior parts [38]. Hybridization technology expands the applicability and functionality of biocomposites, offering broader prospects for their use in advanced engineering and industrial fields. Hybridizing kenaf fibers with synthetic fibers is an effective strategy for enhancing the performance of kenaf fiber-based composites, as synthetic fibers demonstrate superior and more stable mechanical properties compared to kenaf fibers. In addition, the stacking sequence is a critical factor in optimizing the mechanical properties of natural hybrid composites. Symmetrical configurations and optimized stacking sequences can enhance the load distribution and improve the buckling load capacity [128]. It significantly influences the tensile strength and flexural performance of fiber-reinforced composites, with hybrid fiber-reinforced composites generally exhibiting superior ductility than single fiber-reinforced composites [129]. Balakrishnan et al. fabricated hybrid fiber-reinforced composites using a woven kenaf fabric (WK) and unidirectional glass fiber (UG) and found that the WK/UG alternate form provided excellent performance. The results indicated that unidirectional glass fibers can improve the tensile and compressive strengths up to 404 MPa and 440 MPa, respectively, with increasing UG layers in the kenaf composites. It was also found that unidirectional glass fibers can work better with kenaf fibers, achieving an interlaminar shear strength (ILSS) of 34.9 MPa [130].

The extent of biocomposites’ water absorption is closely related to the fiber loading levels, with increased loading typically leading to a higher water absorption capacity [131]. Traditionally, the uptake of water moisture can cause the deterioration of the mechanical properties of fiber-reinforced composites [132,133]. In addition, composites display significant variations in moisture absorption and mechanical properties after aging in different solutions, such as distilled water, seawater, acid solution, and alkali solution [134]. For example, Manral et al. compared the effects of tap water and seawater aging on kenaf/PLA composites. After immersion in tap water and seawater, the tensile strength of the R-kenaf/PLA composites decreased by 39.82% and 49.86%, respectively. The flexural and impact strengths of unidirectional kenaf/PLA composites were reduced by 40.25% and 20.71% in tap water and by 48.07% and 26.03% in seawater [107]. 

The incorporation of nanoparticles at the fiber/matrix interface introduces an interlocking mechanism that enhances the frictional forces between the fibers and the matrix [135]. This process will strengthen the bond between the fibers and the matrix, improve load transfer at the interface, enhance interfacial shear strength and tensile strength, and modify the moisture absorption behavior [23,136]. Taj et al. conducted a study on the water absorption behavior of kenaf/epoxy composites, incorporating nanographene and nanoaluminum oxide as fillers. The results indicated that the combined addition of 6% aluminum oxide and 3% graphene reduced the void content by 77%, leading to a decrease in water absorption from 5.5% and 6.1% to 1.3% and 1.9% in water and seawater, respectively [137]. However, a higher concentration of nanoparticles can increase the brittleness of the composite, which may adversely affect the interfacial strength. Additionally, nanofillers can change the nature of resin; for instance, Malik et al. indicated that the presence of graphene nanoplatelets (GNPs) increased the viscosity and reduced the curing time of epoxy through rheological analysis [138]. This indicates that nanofillers affect the selection of manufacturing methods and available operating time. 

Traditionally, increasing the fiber content enhances the energy absorption capacity of composites to a certain extent [139,140]. Radzi et al. investigated the energy absorption and impact strength of a kenaf fiber-reinforced fiberglass/Kevlar hybrid laminate for boat construction. Their results indicated that both energy absorption and impact strength increased with increasing kenaf fiber content, reaching maximum values of 8.71 J and 85 kJ/m^2^ at a kenaf fiber content of 75% [141]. Moreover, combining natural and synthetic fibers addresses the limitations of natural fibers, achieving a balance between strength and stiffness while improving the overall composite performance [141]. Bhambure et al. created hybrid composites applying kenaf bast fiber and kapok seek fiber, and found that a 50/50 fiber ratio exhibited an excellent impact strength of 46.65 ± 0.01 kJ/m^2^, which surpassed that of the pure kenaf fiber composite. The results revealed that hybridizing kenaf and kapok seed fibers creates a synergistic effect, enhancing the resistance to shear forces and improving the impact performance [57]. Furthermore, compared with kenaf fiber, kapok fiber possesses a larger hollow structure that enables compression under impact, allowing efficient energy dissipation [142].

**Table 7:** Tensile, flexural, impact energy absorption, and moisture absorption values of the kenaf fiber-based composites.

Reinforcement	Matrix	Stacking Sequence	Fiber Ratio	Method	TS (MPa)	TM (GPa)	FS (MPa)	FM (GPa)	EA	WA (%)	Ref.
Kenaf (K)/ Glass (G)	Epoxy	G_4_KG_4_	-	Hand lay-up	94.92	-	-	-	-	-	[108]
Kenaf (K)	PLA	-	30 wt.%	Compression molding	30.25	3.41	71.42	2.85	45.23 J/m	-	[143]
Kenaf (K)	Epoxy	K_5_	-	Vacuum infusion method	79.68	2.21	61.29	4.5	-	-	[144]
Kenaf (K)/ Carbon (C)	Epoxy	CKCKC	-	Vacuum infusion method	210.49	10.6	221.7	30.81	-	-	[144]
Kenaf (K)/ Carbon (C)	Epoxy	KCKCK	-	Vacuum infusion method	134.65	7.95	130	14.02	-	-	[144]
Kenaf (K)/ Carbon (C)	Epoxy	C_2_KC_2_	-	Vacuum infusion method	202.77	10.49	299.31	32.67	-	-	[144]
Kenaf (K)	PLA	-	35 vol%	Compression molding	65.158	3.29	86.956	7.99	-	-	[131]
Kenaf (K)	Unsaturated polyester	K_4_	25.8 wt.%	Hand lay-up	35.78	-	42.91	-	-	-	[145]
Kenaf (K)/ Glass (G)	Unsaturated polyester	KGKG	K:9.63 wt.% G:12.04 wt.%	Hand lay-up	85	-	69.96	-	-	-	[145]
Kenaf (K)/ Glass (G)	Unsaturated polyester	KG_2_K	K:9.94 wt.% G:12.42 wt.%	Hand lay-up	58.75	-	55.62	-	-	-	[145]
Kenaf (K)/ Glass (G)	Unsaturated polyester	GK_2_G	K:9.35 wt.% G:11.70 wt.%	Hand lay-up	77.98	-	59.37	-	-	-	[145]
Kenaf (K)	Epoxy	K_5_	35.4 vol%	Vacuum infusion molding	58.6 ± 5.7	5.2	100.1 ± 2.7	5.7	-	8.0	[146]
Kenaf (K)/ Carbon (C)	Epoxy	KCKCK	K:28.2 vol% C:9.0 vol%	Vacuum infusion molding	178.3 ± 9.1	13.3	188.3 ± 9.9	6.2	-	6.5	[146]
Kenaf (K)/ Carbon (C) fibers	Epoxy	CKCKCKCKC	K:24.6 vol% C:15.8 vol%	Vacuum infusion molding	294.1 ± 10.8	22.4	369.3 ± 9.4	20.0	-	4.2	[146]
Kenaf (K)/ Carbon (C)	Epoxy	KC_2_KC_2_K	K:23.9 V% C:15.4 vol%	Vacuum infusion molding	265.5 ± 7.3	21.0	267.2 ± 9.8	9.2	-	5.7	[146]
Kenaf (K)/ Carbon (C)	Epoxy	C_2_K_3_C_2_	K:25.9 vol% C:16.6 vol%	Vacuum infusion molding	261.8 ± 8.6	18.0	401.0 ± 11.5	29.3	-	4.3	[146]
Kenaf (K) fiber	Epoxy	-	35.9 vol%	Vacuum infusion molding	55.6	8.4	111.9	5.4	-	5.2	[138]
Kenaf (K)/ UD-Glass (G)	Unsaturated polyester	KGKGKGK	-	Hand lay-up and Compression molding	404.54	25.54	-	-	-	-	[130]
Kenaf (K)/ UD-Glass (G)	Unsaturated polyester	KGKGKGK	-	Pultruded method	410.6	26	-	-	260 kJ/m^2^	-	[147]
Kenaf (K)	Unsaturated polyester	-	6%	Hand lay-up	40.6	-	54.02	2.25	44.95 kJ/m^2^	-	[57]
Kenaf (K)/ Glass (G)	Epoxy	GKGKG	30%	Hand lay-up	147.64	3.9	188.99	10.9			[148]
Kenaf (K)/ Glass (G)	Epoxy	KGKGK	30%	Hand lay-up	121.45	2.96	155.22	5.74			[148]
Kenaf (K)/ Glass (G)/ Tea leaf (T)	Epoxy	GKGTGKG	K:25% G:10% T:5%	Compression molding	70.81	-	181.08	-	96 kJ/m^2^	-	[149]
Kenaf (K)	Epoxy	K_5_	21.91%	Hand lay-up	31.9	2.87	43.1	3.85	3 J	-	[125]
Kenaf (K)/ Areca (A)	Epoxy	AKAKA	28.45%	Hand lay-up	8.76	0.47	15.7	1.35	15 J	-	[125]
Kenaf (K)/ Areca (A)	Epoxy	KAKAK	27.28	Hand lay-up	13.98	1.39	26.9	4.21	8 J	-	[125]
Kenaf (K)	PLA	K_5_	40%	Hot compression process	61	5.4	62	2.7	49 kJ/m^2^	-	[150]
Kenaf (K)/ Polyester (P)	PLA	PK_3_P	K:23.5% P:16.5%	Hot compression process	36	5.4	89	5.4	63 kJ/m^2^	-	[150]
Kenaf (K)/ Polyester (P)	PLA	P_2_KP_2_	K:7.7% P:32.3%	Hot compression process	103	0.7	80	5.5	83.6 kJ/m^2^	-	[150]
Kenaf (K)	Epoxy	-	30%	Vacuum infusion molding	70.96	2.09	59.04	3.77	-	25.49	[132]
Kenaf (K)	Epoxy	-	40%	Vacuum infusion molding	76.67	2.31	61.24	4.2	-	30.46	[132]
Kenaf (K)/ Flax (F)	Epoxy	FKF	-	Hand lay-up	41.06 ± 1.83	2.6 ± 0.2	64.13 ± 6.29	3.80 ± 0.2	-	-	[151]
Kenaf (K)/ Flax (F)	Epoxy	KFK	-	Hand lay-up	44.4 ± 1.83	2.39 ± 0.1	64.94 ± 7.68	4.44 ± 0.3	-	-	[151]
Kenaf (K)/ Jute (J)	Epoxy	JKJ	-	Hand lay-up	40.66	3.27	57.2	3.24	-	-	[129]
Kenaf (K)/ Jute (J)	Epoxy	KJK	-	Hand lay-up	43.21	3.60	75.57	4.62	-	-	[129]
Kenaf (K)/ Glass (G)	Epoxy	G_2_K_2_G_2_	-	Vacuum-bagging process	42.69	-	158.42	-	-	-	[152]
Kenaf (K)/ Glass (G)	Epoxy	KG_4_K	-	Vacuum-bagging process	88.08	-	71.63	-	-	-	[152]
Kenaf (K)/ Jute (J)/ Glass (G)	Epoxy	K_2_JKG_2_	-	Vacuum-bagging process	41.613	-	243.86	-	-	-	[152]

Note: (a) Abbreviations are defined as follows: TS for tensile strength, TM for tensile modulus, FS for flexural strength, FM for flexural modulus, EA for energy absorption, WA for water absorption at saturation, and PLA for polylactic acid. (b) In the stacking sequence column, the numbers represent the number of fiber layers; for instance, G_4_KG_4_ indicates a sequence configuration of four layers of glass, one layer of kenaf, and four layers of glass.

## 6. Exploring Kenaf Fiber Composites for Marine Applications

The growing impact of global environmental challenges and rising awareness of sustainable resource usage have driven advancements in research focused on creating next-generation biodegradable and environmentally friendly materials [153,154]. Natural fibers are generally characterized by their non-toxic, hypoallergenic, and biodegradable properties, making them highly suitable for bio-health applications and promoting environmental sustainability [155]. However, plant fiber composites tend to absorb moisture when exposed to water or humid environments, leading to a deterioration in their mechanical properties and a potential risk of premature failure during prolonged environmental exposure [107]. In order to enhance the durability of kenaf fiber-reinforced composites, researchers have performed several experiments to increase their moisture resistance, as shown in Table 8. The results indicated that alkali treatment, surficial modification technology, hybridizing with synthetic fiber, and integrating nanofillers are effective methods for reducing water uptake.

**Table 8:** The works related to decreasing the moisture absorption of kenaf composites.

**Fiber-Matrix**	**Applied Methods**	**Results**	**Ref** **.**
Abaca (A)/Kenaf (K)/Carbon (C)-Epoxy	Use alkali-treated natural fibers; Change stack forms, including CKKKC, CAAAC, CAKAC, and CKAKC.	Alkali-treated plant fibers reduced the diffusion coefficient of composite, CKKKC, by 30.5% (from 4.047 × 10^−13^ m^3^/s to 2.813 × 10^−13^ m^3^/s); Changing stack order can decrease the diffusion coefficient of CKAKC by 15.98% compared to CAAAC (from 3.799 × 10^−13^ m^3^/s to 3.192 × 10^−13^ m^3^/s).	[156]
Kenaf (K)-Epoxy	Control fiber content in composites with 30 vol%, 40 vol%, and 50 vol%.	Under saturated conditions, the water absorption radio of composites with varying fiber contents are measured as 25.49% for 30 vol%, 30.46% for 40 vol%, and 43.33% for 50 vol%; Water uptake ratio is influenced by the fiber content.	[132]
Kenaf (K)/Glass (G)-Epoxy	Treat kenaf fibers with 0.5% NaOH for 10 minutes; Reinforce composites with glass fiber and 0.5% and 1.0% graphene.	The hybrid kenaf/glass fiber reinforced 1% graphene nanoparticle composite displayed less water absorption than pure kenaf composite, and the reduction is 58.33% (from 5.28% to 2.20%); Both hybridizing kenaf fiber with glass fiber and adding nanoparticles can reduce the water uptake ratio and can reduce the ratio by 53.97% and 9.47%.	[157]
Kenaf (K) fiber	Treat kenaf fibers with 5% NaOH for 3 hours.	Alkali-treated kenaf fibers reduced the water uptake by 15.6% compared to the untreated ones.	[158]
Kenaf (K)-PLA	Treat kenaf fibers with 5% NaOH for 3 hours; Integrate 1 wt.%, 2 wt.%, and 3 wt.% montmorillonite clay filler into composites.	Integrating montmorillonite clay can increase the water repulsion; Hybridizing 3 wt.% montmorillonite clay decreased the water uptake from 2.18% to 1.45% and 9.45% to 6.18% at 3 and 30 days compared to the 0% nanofiller-added composite.	[159]

**Figure 10:**
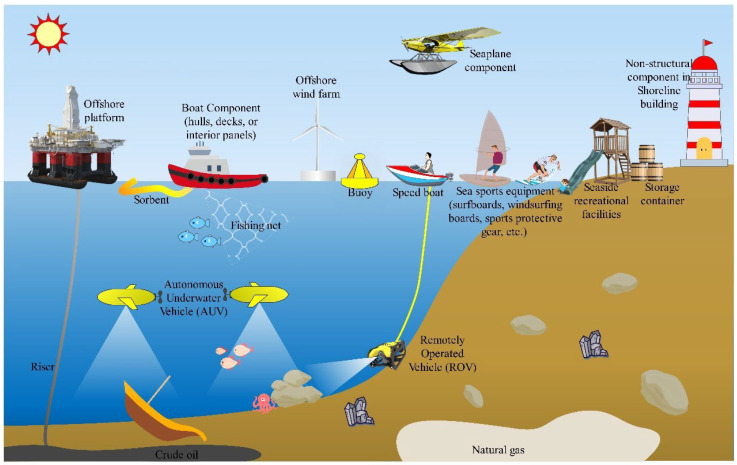
Applications of natural fiber-reinforced composites in the marine environment.

**Figure 11:**
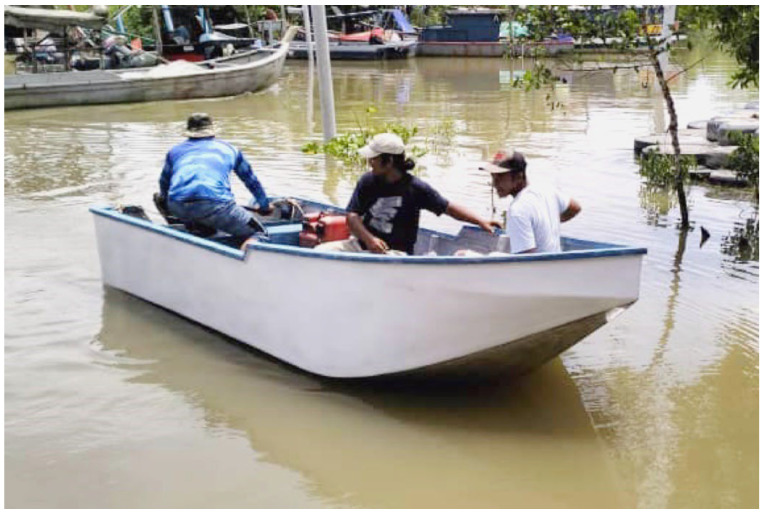
Kenaf fiber composite boat [164].

While natural fiber materials have limitations, including stability, hygroscopicity, and weatherability, many researchers remain convinced that they provide a significant alternative to synthetic fibers. After rigorous performance testing, plant fiber-based composite products have gained widespread use in the aerospace, land transportation, and marine industries [160,161]. Traditionally, plant fiber-reinforced composites are often used as internal components in dry working conditions, mainly because the hydrophilicity of plant fibers can lead to the degradation of the mechanical properties of the composite materials. However, these biocomposites have a highly advantageous influence on marine applications owing to their excellent lightweight characteristics and environmentally friendly performance. Natural fiber-reinforced biocomposites are increasingly utilized in various marine applications, such as boat hulls, decks, surfboards, buoys, sorbents, risers, and other maritime components, as illustrated in Figure 10. Figure 11 shows a small boat made of kenaf fiber. Although kenaf fiber is widely recognized as an important cellulose fiber resource and has been extensively studied, its application in marine environments remains relatively scarce. Therefore, further research is needed to advance our understanding and exploration of this field.

For marine applications, addressing the hygroscopic effects of natural fibers is an unavoidable challenge. The harsh conditions of seawater have more severe adverse effects on the tensile, flexural, and impact properties of kenaf-based composites [107]. The hygroscopic behavior of plant fiber is determined by a combination of intrinsic factors, such as fiber type and chemical composition, and environmental conditions, such as ambient humidity and temperature [162]. Converting the inherent hydrophilicity of natural fibers to hydrophobicity is critical for modifying the physical, mechanical, and thermal properties of natural fiber-reinforced composites [163]. 

During seawater immersion, seawater molecules first penetrate the internal structure of the composite through surface microcracks or capillary action. Then, these molecules diffuse further into the material, penetrating the fiber-matrix interface and internal fibers. In addition, greater moisture uptake induces plasticization of the matrix, decreasing the mechanical properties of the composites [165]. Furthermore, seawater infiltration accelerates the degradation of natural fiber-reinforced composites, often leading to fiber-matrix debonding, delamination, cracking, swelling, dimensional and morphological instability, salt deposition, and corrosion, all of which can negatively impact the structural integrity and safety [166]. 

Singh et al. aged kenaf/HDPE composites in seawater for six months and found that while the mechanical properties of the composite decreased with extended soaking time, the hardness of the material increased. The researchers attributed this to the accumulation of seawater particles such as K, Ca, Na, Cl, Mg, and F on the composite surface, which had higher hardness than the original composite [167]. Furthermore, these particles can accumulate within the kenaf fibers, obstructing the pathways of seawater in the kenaf/HDPE composite, thereby reducing the composite’s seawater absorption capacity [168]. However, seawater exhibits strong degrading and corrosive effects, primarily due to its high concentration of chloride ions. Therefore, surface coatings or modifiers should be effective methods for protecting natural fiber composites and enhancing their resilience in wet environments [167]. 

Nanomaterials can modify the hygroscopic properties of kenaf-based composites, thereby improving their moisture resistance [169]. Nanoparticles possess a large specific surface area, increasing the interfacial positions for the interaction between the polymer matrix and filler [170]. The formation of these interfaces plays a crucial role in modifying the properties of composites. Taj et al. successfully reduced the porosity and seawater absorption of kenaf/epoxy composites by optimizing the ratio of alumina to graphene nanoparticles [137]. Raj et al. incorporated 0%, 2%, 4%, 6%, and 8% graphene nanofiller in kenaf/epoxy and discovered that the composite with 8% graphene content demonstrated the lowest water content (5.13%), which is a 51.42% decrease relative to the graphene-free composite [171]. Increasing the number of nanoparticles significantly reduces the water absorption capacity of the composites. In addition, a similar result was observed by Palmiyanto et al., who added 4%, 8%, and 12% microcrystalline cellulose (MCC) nanoparticles to glass and kenaf fiber-reinforced phenolic resin composites and found that 12% MCC nanoparticles exhibited the lowest seawater absorption [172]. Furthermore, Liu et al. found that nano SiO_2_ effectively forms a molecular chain interlocking mechanism and polar interactions between plant fibers and the matrix in composites, which inhibits water molecule diffusion, improves water resistance, and preserves the dimensional stability of the composite materials [23]. In their experiment, they reported that the influence of nano SiO_2_ in composites can reduce the water absorption rate by 34.52% and the thickness expansion rate by 60% [23]. Integrating nanoparticles to a certain proportion in fiber-reinforced composites can significantly decrease water uptake, whether plant fiber-based or synthetic fiber-based composites [173,174,175]. Therefore, applying nanoparticle technologies is a suitable approach for enhancing the properties of kenaf fiber-based composites in marine environments.

## 7. Conclusions

The kenaf plant is cultivated extensively as a commercial crop and has several advantages, such as a short growth cycle, adaptability to various environments, and resistance to pests and diseases. The plant is widely used in the food and medicine industries. In addition, the thick phloem layer of the kenaf stem enables a high yield of kenaf bast fiber. The high cellulose content of the fiber imparts excellent mechanical properties, making it a viable and sustainable alternative to synthetic fibers for applications in the aerospace, automotive, and marine sectors. Furthermore, as a renewable and biodegradable resource, kenaf fiber can reduce the carbon footprint and mitigate ecological harm. This manuscript provides an overview of the properties and potential applications of bast fiber and kenaf fiber-based composites in seawater environments. 

Advanced manufacturing techniques, including mature and systematic experimental methods, play a pivotal role in advancing the application of fiber-reinforced composites. These techniques significantly accelerate the transformation of kenaf fibers into composite materials, promoting the modification of agricultural products to industrial products and enhancing their value. In addition, numerous studies have demonstrated that kenaf fiber exhibits excellent synergy and compatibility with other fibers. Composite laminates combining kenaf fibers with different fibers, particularly synthetic ones, exhibit excellent mechanical properties. Hybridization technology can significantly improve the performance of kenaf fiber-based composites, thereby expanding their potential application scope, especially in oceans.

However, cellulose fibers are extensively used in marine-related fields, including boat construction, marine sports equipment, coastal engineering, offshore engineering, and environmental protection. The application of kenaf fiber-based composites in marine environments remains in the primary stage, with relatively few cases reported. Further detailed explorations are needed, especially in developing effective strategies to reduce property fluctuations in kenaf fiber-based composites. The use of these composite materials in marine applications continues to face several challenges and research gaps, such as:

- The impact of seawater absorption and UV radiation on the mechanical, thermal, physical, and chemical properties of the composites;
- The debonding effects of interfacial bonding between kenaf fibers and matrix materials in seawater environments;
- The long-term effects of seawater exposure on the degradation, corrosion, and durability of kenaf fiber-based composites.

In conclusion, the development of innovative manufacturing techniques, efficacious surface treatment technologies, and advanced testing methods can provide technical support for expanding the application field of kenaf fiber-reinforced composites. Furthermore, these biocomposites will exhibit greater resistance in harsh marine environments and expand their utilization in the marine industry, with the enhancement of mechanical properties (such as tensile, compressive, flexural, and impact resistance) and environmental durability (such as water aging, thermal aging, ultraviolet aging, and hygrothermal aging).

## Figures and Tables

**Table 1:** Global production of kenaf and related fibers (2017/18–2021/22, Unit: Kilotons) [4].

Location	2017/18	2018/19	2019/20	2020/21	2021/22
I. Developing countries	202.6	198.6	204.6	200.3	195.7
a. Far East	155.3	151.3	157.7	150.2	146.7
1. India	100	100	100	100	100
2. China	50	45.97	52.37	45.03	41.43
3. Indonesia	3.3	3.3	3.4	3.3	3.3
4. Pakistan	1	1	1	1	1
5. Vietnam	0.5	0.5	0.5	0.3	0.4
6. Thailand	0.2	0.2	0.2	0.3	0.3
7. Cambodia	0.2	0.2	0.2	0.3	0.3
b. Latin America and the Caribbean	26	26.1	25.6	28.5	27.3
1. Cuba	11.9	12	11.8	11.8	11.8
2. Brazil	2.8	2.9	2.6	5.5	4.4
3. Other	11.3	11.2	11.2	11.2	11.1
c. Africa	15.5	15.4	15.5	15.6	15.7
d. Near East	5.7	5.7	5.8	6	6
II. Developed countries	6.8	6.8	6.9	6.8	6.8
Total (I + II)	209.4	205.4	211.5	207.1	202.5

**Table 2:** Chemical components of kenaf fiber.

No.	Cellulose (%)	Hemicellulose (%)	Lignin (%)	Pectin (%)	Moisture (%)	Wax (%)	Ash (%)	Ref.
1	44–57	21	15–19	2	-	-	-	[3]
2	45–57	21.5	8–13	-	-	-	-	[4]
3	66.9	14.98	6.85					[37,51]
4	31–57	21.5–23	15.0–19	3–5	-	-	-	[37,52]
5	65.7	17.8	6.0	-	-	-	-	[37,53]
6	45–57	8.0–13.0	21.5	0.6	6.2–12	0.8	2–5	[54]
7	53–57	15–19	-	5.9–9.3	7–10	-	-	[55]
8	69.2	27.2	2.8	-	8–12	-	0.8	[56]
9	44–57	22–23	15–19	-	-	-	0.8	[57]
10	72	20.3	9	-	-	-	-	[58]
Range	31–72	13.59–23	2.8–20.1	0.6–9.3	6.2–12	0.8	0.8–5	

**Table 3:** Common physical and mechanical properties of kenaf fiber.

S. No.	Tensile Strength (MPa)	Tensile Modulus (GPa)	Elongation at Break (%)	Density (g/cm^3^)	Diameter (μm)	Specific Strength (N·m/g)	Specific Stiffness (N·m/g)	Ref.
1	223	15	5.7	-	140	-	-	[55]
2	350–600	40	2.5–3.5	1.2	50–60	291.7–500	2083.3–2916.7	[66]
3	284–930	21–60	1.6	1.45	-	195.9–641.4	1103.4	[67]
4	284–800	21–60	1.6	1.4	-	202.9–571.4	1142.9	[37,64]
5	350–600	40	2.5–3.5	1.5	-	233.3–400	1666.7–2333.3	[37,68]
6	-	-	1.9–4.8	-	80–144.8	-	-	[50]
7	-	-	-	1.4	-	-	-	[56]
8	223–1191	11–60	1.6–4.3	1.5	-	148.7–794	1066.7–2866.7	[69]
9	290–950	50	-	1.2	-	241.7–791.7		[57]
Range	223–1191	11–60	1.6–5.7	1.2–1.5	50–144.8	148.7–794	1066.7–2916.7	

**Table 4:** Comparison of the kenaf fiber retting methods [72,73,74,75,76].

Retting Method	Principle	Advantages	Disadvantages
Mechanical	Separates fibers from bast layers via mechanical force.	Fast and efficient; No chemicals or biomaterials required; Short and coarse fibers.	High fiber breakage; Incomplete separation; High energy consumption.
Chemical	Dissolves lignin and pectin using acids, alkalis, or other chemical solutions.	Separation is thorough and efficient; Rapid processing; Soft, long, and fine fibers.	Environmental pollution; High cost (reagents/energy); May damage fibers.
Biological	Uses microbes or enzymes to degrade lignin and pectin.	Eco-friendly; Minimal fiber damage; Low energy use, Uniform, long, and high-strength fibers	Slow process (days to weeks); Requires controlled operating conditions; High enzyme costs.
Physical	Separates fibers from bast layers in natural circumstances.	Low cost; Simple method; Coarse, stiff fibers.	Time-consuming (weeks to months); Climate-dependent; Inconsistent quality.

**Table 5:** Routine testing items of composite materials for mechanical and physical properties [105].

S. No.	Test Item	ASTM Standard	Brief Description of the Test	Results Data
1	Tensile test	D3039	Assess the in-plane tensile characteristics of polymer matrix composites reinforced with high-modulus fibers to understand their performance under specific loading conditions; The composite materials examined are restricted to continuous and discontinuous fiber-reinforced formation; The sample shape is a rectangle.	Tensile strength; Tensile strain; Tensile modulus elasticity; Poisson’s ratio; Transition strain.
2	Tensile test	D638	Determine the tensile properties of both unreinforced and reinforced plastics; The standard specimen shape is dumbbell-shaped.	Tensile strength; Tensile strain; Other tensile properties.
3	Flexural test	D790	Assess the flexural properties of both unreinforced and reinforced plastics; Can be applied for testing both rigid and semirigid materials; The test samples are rectangular cross-sections; Utilize the three-point loading mode; The ratio of support span-to-thickness is 16:1.	Flexural strength; Flexural stress at break; Flexural stress-strain relationship; Flexural modulus of elasticity.
4	Flexural test	D6272	Determine of flexural properties of unreinforced and reinforced plastics; Apply for rigid and semirigid materials; Common employed for materials not fail within the strain limits (5.0% strain) imposed by the method D790; Utilize the four-point loading system.	Flexural strength; Flexural strain; Other Flexural properties.
5	Flexural test	D7264	Determine the flexural properties of polymer matrix composites; Two Loading Procedures: (1) Three-point center loading; (2) Four-point loading; The standard support span-to-thickness ratio is 32:1.	Flexural Strength; Flexural Stiffness; Load/Deflection Behavior.
6	Compression test	D695	Determines the compressive properties of unreinforced and reinforced rigid plastics, including high-modulus composites, under low, uniform strain or load rates; Test specimens are standard-shaped, with applications for a composite modulus up to 41,370 MPa.	Compressive strength; Modulus of elasticity; Yield stress; Deformation beyond the yield point.
7	Compression test	D3410/D3410M	Determines the in-plane compressive properties of polymer matrix composite materials reinforced by high-modulus fibers; Uses a shear loading approach with compressive force applied through shear at wedge grip interfaces; Provides compressive property data.	Ultimate compressive strength; Ultimate compressive strain; Compressive modulus of elasticity (linear or chord); Poisson’s ratio in compression; Transition strain.
8	Compression test	D6641/D6641M	Determines compressive strength and stiffness properties of polymer matrix composites using combined loading compression with end- and shear loading; Suitable for general, balanced, symmetric composites; Allows testing of tabbed and un-tabbed specimens depending on material type.	Ultimate compressive strength; Ultimate compressive strain; Compressive modulus of elasticity (linear or chord); Poisson’s ratio in compression.
9	Water absorption test	D570	Determines the relative rate of water absorption by plastics when immersed; Applicable to cast, hot-molded, cold-molded, homogeneous, and laminated plastics in rod, tube, and sheet forms (0.13 mm thickness or greater); Used to assess the effect of water absorption.	Water absorption rate; Saturation time.
10	Density and specific gravity	D792	Determines the specific gravity (relative density) and density of solid plastics in various forms; Two methods: Test Method A (in water) and Test Method B (in liquids other than water).	Density; Specific gravity.
11	Apparent density test	D1895	Measures apparent density, bulk factor, and pourability of plastic materials such as powders and granules.	Apparent density; Bulk factor; Pourability.
12	Void content of reinforced plastics	D2734	Measures the void content in reinforced plastics or composites; Useful for quality estimation of composites.	Void content.
13	Izod Pendulum Impact Resistance	D256	Measures the ability of plastic materials to resist fracture under impact conditions; Tested with a pendulum impact device, recording the energy absorbed to break a sample.	Energy Absorption.
14	Charpy Impact Resistance	D6110	Measures the resistance of plastics to breakage under a single impact using a pendulum-based hammer on a notched specimen; The energy required to break the specimen is recorded, reflecting properties like fracture initiation and propagation and includes factors like vibration and bending.	Energy Absorption.
15	Damage Resistance of Fiber-Reinforced Polymer Matrix Composites	D7136/D7136M	Measures the damage resistance of fiber-reinforced composite laminates to drop-weight impacts; Assesses how factors such as stacking sequence, fiber treatment, fiber volume, processing, and environmental factors affect damage resistance; Provides data on impact damage, including depth, dimensions, and force-time characteristics; Used to compare materials, support design, and impart damage for further tolerance tests.	Damage Size and Type.

**Table 6:** The influence of alkali treatment on the mechanical properties of kenaf fibers and their composites.

Fiber-Matrix	Alkali Solution	Treatment Parameters	Highlight RESULTS	Ref.
Kenaf-Epoxy	5%(*w*/*v*) NaOH	24 h 28°C	The tensile strength of kenaf fiber increased by 81% and reached 585.1 MPa; The tensile strength and flexural strength of kenaf(treated)/epoxy composite increased by 9.57% and 17.25%, reached 61.8 MPa and 221.6 MPa.	[117]
Kenaf-Epoxy	6 wt.% NaOH	24 h room temperature	The composite made by alkali-treated kenaf fiber displayed better thermal stability compared to untreated ones, according to the data of TGA; Treated kenaf fibers decreased composites’ tensile strength and modulus by 34.65% and 11.92% compared to applying untreated ones.	[116]
Kenaf/Glass-Epoxy	2 M NaOH	4 h room temperature	The flexural strength and modulus and the impact-absorbed energy of the treated kenaf composites increased by 52%, 46%, and 83%, respectively, compared to untreated ones.	[118]
Kenaf fiber	6 wt.% NaOH	4 h room temperature	The tensile strength of kenaf fiber is 282.7 MPa, increasing by 169.13% compared to the untread one, while the value of elongation at break and Young’s modulus increased by 41.31% and 158.52% also.	[119]
Kenaf-Epoxy	6 wt.% NaOH 6 wt.% KOH	8 h	The tensile, flexural, and impact strengths of NaOH-treated kenaf fiber composites are 58.34 MPa, 55.34 MPa, and 16.69 kJ/m^2^, respectively; The tensile, flexural, and impact strengths of KOH-treated kenaf fiber composites are 53.69 MPa, 53.44 MPa, and 15.24 kJ/m^2^, respectively.	[120]

## Data Availability

No new data were created or analyzed in this study.
